# Molecular survey of Zika virus in the animal-human interface in traditional farming

**DOI:** 10.3389/fvets.2022.1057686

**Published:** 2022-11-25

**Authors:** Laura Ivone Lopez-Apodaca, Heliot Zarza, Emily Zamudio-Moreno, Daniel Nuñez-Avellaneda, Carlos Marcial Baak-Baak, Guadalupe del Carmen Reyes-Solis, Torres-Chablé Oswaldo Margarito, Ingris Peláez-Ballestas, David Roiz, Gerardo Suzán, Benjamin Roche, Carlos Ignacio Machain-Williams

**Affiliations:** ^1^Laboratory of Arbovirology, Regional Research Center Dr. Hideyo Noguchi, Autonomous University of Yucatan, Mérida, Mexico; ^2^Department of Environmental Sciences, Lerma Unit Metropolitan Autonomous University, Mexico City, Mexico; ^3^Direction Adjunt of Technological Development, Liaison and Innovation-National Science and Technology Council, Mexico City, Mexico; ^4^Laboratory of Tropical and Vector-Borne Diseases DACA-UJAT, Academic Division of Agricultural Sciences, Juarez Autonomous University of Tabasco, Villahermosa, Mexico; ^5^Rheumatology Unit, Hospital General de México Dr. Eduardo Liceaga, Mexico City, Mexico; ^6^Infectious Diseases: Vector, Control, Genetic, Ecology and Evolution (MIVEGEC), Univ. Montpellier, IRD, CNRS, Montpellier, France; ^7^International Laboratory Ecosystem, Biological Diversity, Habitat Modifications, and Risk of Emerging Pathogens and Diseases in Mexico (ELDORADO), Mérida, Mexico; ^8^Laboratory of Disease Ecology and One Health, Department of Ethology and Wildlife, Faculty of Veterinary Medicine and Zootechnics, National Autonomous University of Mexico, Mexico City, Mexico

**Keywords:** flavivirus, Zika virus, arbovirus, Yucatan (Mexico), human-animal interface, backyard animal rearing, mosquitoes, vulnerable communities

## Abstract

Backyard animal husbandry is common in rural communities in developing countries and, given the conditions in which it occurs, it can increase the risk of disease transmission, such as arboviruses. To determine the presence of the Zika virus (ZIKV) and abundance of its arthropod vectors we evaluated the socioeconomic implications involved in its transmission in two highly vulnerable Mayan communities in the state of Yucatan that practice backyard farming. An analytical cross-sectional study was carried out throughout 2016 to understand socioeconomic variables and seasonal patterns in mosquito populations. We selected 20 households from each community. Social exclusion indicators were analyzed, human and domestic animals were sampled, and mosquitoes were collected and identified. Four out of eight indicators of social exclusion were higher than the reported national averages. We captured 5,825 mosquitoes from 16 species being *Culex quinquefasciatus* and *Aedes aegypti* the most abundant. The presence of chickens and human overcrowding in dwellings were the most significant factors (*P* = 0.026) associated with the presence of *Ae. aegypti*. Septic tanks (odds ratio = 6.64) and chickens (odds ratio = 27.41) in backyards were the main risk factors associated with the presence of immature states of *Ae. aegypti* in both communities. Molecular analysis to detect ZIKV was performed in blood samples from 416 humans, 1,068 backyard animals and 381 mosquito pools. Eighteen humans and 10 pig pools tested positive for ZIKV. Forty-three mosquito pools tested positive for flavivirus. Ten of the 43 pools of positive mosquitoes were sequenced, corresponding 3/10 to ZIKV and 1/10 to Dengue virus type 2. The findings obtained indicate the continuous circulation of Flavivirus (including ZIKV) in backyard environments in vulnerable communities, highlighting the importance of studying their transmission and maintenance in these systems, due that backyard animal husbandry is a common practice in these vulnerable communities with limited access to health services.

## Introduction

Emerging diseases are increasing in number along with human population growth. The anthropic activities have resulted in land-use changes, causing deforestation and biodiversity loss to increase trade and intensive farming (1). Intensive farming and livestock expansion have been clearly related with changes in land use, being the latter a relevant driver for disease emergence or re-emergence (2). The relationship between intensive farming activities and the emergence of infectious diseases has been ascertained as the main driver transforming ecosystems by increasing interactions among humans, animals, and their pathogens (3). However, the relationship between traditional farming and emerging diseases has been less studied, even though in the tropics, especially in developing countries, traditional farming is practiced by more than half million farmers who are at risk of contracting potential zoonotic diseases (4, 5). Traditional farming is a common practice in rural communities in most developing countries, more than 75% of rural families in Mexico practice this livestock production system in their backyards (6). This activity represents a critical income for rural populations, either for self-consumption or community trade, and is characterized by low technification, lack of proper infrastructure, improvised animal management and an insufficiency of appropriate public health programs and diseases surveillance, resulting potentially in human-animal pathogen exchange and pathogen spill-over (7).

Arthropod-borne viruses, also known as arboviruses, are currently one of the most relevant public health issues in the field of emerging infectious diseases, and many of those viruses transmitted by mosquitoes are of zoonotic origin, producing severe infections in humans and animals (8, 9). The Flaviviridae family englobes a large number of positive sense RNA enveloped arboviruses which circulate between humans and animal reservoirs and amplifiers (10). Zika virus (ZIKV) belongs to the genus *Flavivirus*, taxonomically is classified in the Ntaya virus group (11). Viruses in this group maintain an enzootic cycle with the potential to infect humans (12, 13). ZIKV was first detected in the Zika forest in Uganda in the blood of a febrile non-human primate (14). ZIKV infections were first classified as mild, non-life-threatening infections, until its reemergence in the pacific islands and Brazil where it was associated with cases of microcephaly. Later, further virus analysis from affected patients identified the virus strain as the Asian genotype (15). The main mechanism of ZIKV transmission is the bite of an infected mosquito, with *Ae. aegypti* being the main vector in the Americas outbreak. However, vector competence and surveillance studies of ZIKV in different parts of the world have demonstrated the permissiveness of *Cx. quinquefasciatus* to this virus under experimental conditions as well as in naturally infected mosquitoes caught in the field (16, 17).

Since its first report in America, ZIKV quickly spread throughout the continent (18), causing a severe burden on public health. Clinical manifestations of Zika virus infections in humans include rash, fever, myalgia, headache, conjunctivitis, retro-orbital pain, edema, pruritus, and fatigue (14). Also, the presence of nervous system afflictions has been reported in children and adults without microcephaly syndrome after Zika infection (19). Undoubtedly, the most severe affliction of all is microcephaly, which can be seen not only as a major public health concern, but also, carries a high economic impact (20) and social stigma for the families of affected newborns in endemic regions of the Americas (15, 21). Because of the zoonotic nature of Ntaya virus group (10, 13), the capability of Zika virus to infect domestic animals (22), and the broad immunological cross-reactivity between flaviviruses (23), molecular survey was used as an easy, inexpensive alternative, to screen Zika virus spread in the animal-human interface where backyard farming is practiced as a subsistence activity.

## Materials and methods

### Study sites

**Figure 1:**
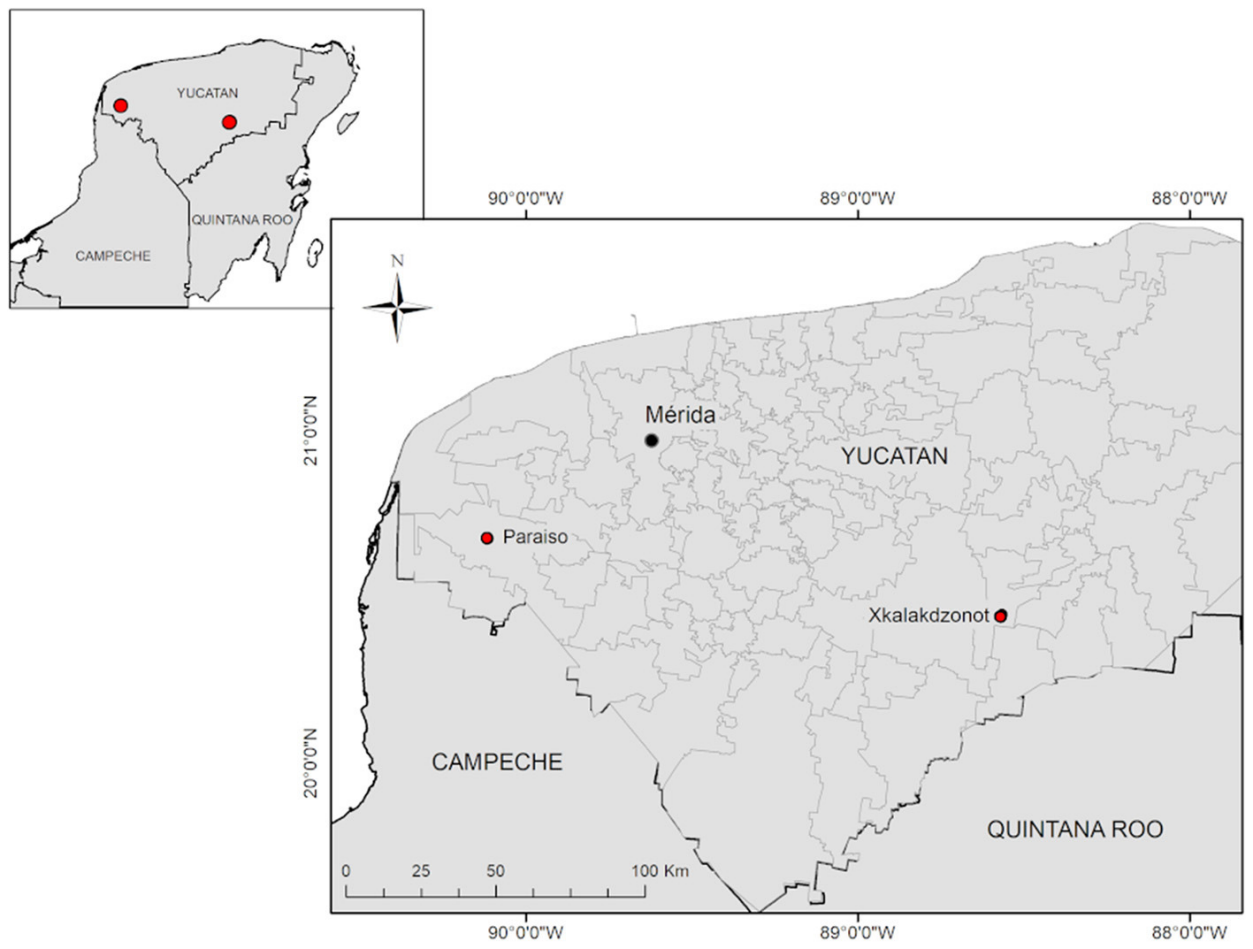
Map of the geographical location of the Mayan communities studied in Yucatan, Mexico. Paraiso (PSO) located in the municipality of Maxcanú, and Xkalakdzonot (XKT) located in the municipality of Chankom.

The study was conducted in two rural Mayan communities (Figure 1), classified with a high level of social marginalization, traditional farming activities, and a historical presence of arbovirosis associated with febrile-like illness located in the state of Yucatan, Mexico (2,097 175 inhabitants, 16% living in rural areas) (24).

We selected the community of Xkalakdzonot (XKT), which belongs to the municipality of Chankom and has a population of 789 inhabitants (25). This municipality is located in the center-south of the state, 123 km from Mérida, the state capital. The rural community of Paraíso (PSO), which belongs to the municipality of Maxcanu and has a population of 656 inhabitants. PSO is located in the western part of the state, 65 km from the state capital (25).

### Communities' characteristics

More than 95% of the population of both communities is of Mayan descent, and more than 70% of the inhabitants speak Mayan language. Most houses are built out of concrete, and to a lesser extent, with wooden walls and palm leaf roofs. The houses have extensive patios (~2,000 m^2^) surrounded by abundant vegetation patches of low deciduous forest. Inhabitants live in houses with concrete floors; some rooms are built out of concrete and cement, or built with stones, wooden poles, and palm leaf roof. Also, some of the house rooms are used simultaneously as bedrooms, kitchens, storerooms, or shops. In both communities it is common to observe waste accumulated in backyards, mostly food scraps, cardboard, plastic containers and old electronic appliances and furniture. Backyards are occupied by local flora where trees serve as a source of food such as citrus and forage to feed the animals. Among the most common native trees found in these communities are, breadnut (*Brosimum alicastrum*) and Leucaena bush (*Leucaena leucocephala*).

### Study design

An analytical cross-sectional study was carried out by dividing the communities into four quadrants, and five families per quadrant were randomly invited to participate. Twenty enrolled families per town (40 total) participated in the study for 1 year. The sampling period was during the rainy season (May-October) and the dry season (January-April) of 2016.

A survey was conducted in each household to determine socioeconomic aspects of both communities (education, housing, and monetary income), their livestock inventory, and their knowledge about mosquito-borne diseases and their transmission. To assess social marginalization, the National Population Council of Mexico (CONAPO) designated eight forms of exclusion (percentages): illiterate population ≥15 years old, population ≥15 years old without completing primary school, dwellings without availability of drainage or toilets, dwellings without availability of electricity, dwellings without availability of piped water, crowding >2 persons per room, houses with dirt floors, and population earning <2 minimum wages per day (24). These forms of exclusion were used to compare what was observed in the communities studied with the reported national averages. All interviews were conducted with the support of a Mayan language translator.

### Ethics statement

The studies involving human participants were reviewed and approved by the Research Ethics Commission under the supervision of Hospital General de Mexico “Dr. Eduardo Liceaga” (Registry key: DI/14/ 404D/05/050). The patients/participants provided their written informed consent to participate in this study. All people diagnosed with a disease received specialized care in the community and steps were taken to ensure continuity of their care in the health system.

The animal study was reviewed and approved by the Autonomous University of Yucatan (CB-CCBA-I-2017-006). Written informed consent was obtained from the owners for the participation of their animals in this study.

### Animal population

XKT has a higher number of pigs, chickens, and turkeys. Small pens (2 x 3 mts) are used to confine the animals. Pens are made of autochthonous materials such as wood with palm leaf roofing and in some cases concrete walls. Around 20% of the housing construction material is made of recycled materials such as zinc laminated roofs or cardboard sheets. Most of the flocks (chickens and turkeys) forage freely around the houses and are confined during the nights, whereas pigs are confined in around 50% of the houses and only confined during farrowing and weaning. XKT is prominent for pig inventory with 95% of the households rearing at least one, whereas poultry is present in 80% of the homes.

In the community of PSO, there is a predominately poultry population (98% of households), whereas only 5% of the population intermittently produces pigs. Ninety percent of the population in both communities use sinks and troughs as containers for animal feed and water.

### Blood collection

Blood samples were collected from apparently healthy humans and animals once the informed consent was signed and permits from family heads and animal owners allowed the intervention. Human blood samples were obtained using 5 ml vacutainer (red) tubes directly from the brachial or cephalic vein of the human participants from each household. Most participants were asymptomatic at the time of the study. An aliquot of 0.5 ml of blood was placed in RNA stabilizer to preserve viral genetic material. Blood from pigs was drained from the anterior cava vein using a 10 ml vacutainer (red) tube. Direct ulnar vein puncture from broilers and turkeys yielded around 1.5 and 2 ml of blood, respectively by using 3 ml syringes. All samples were kept in a cold chain until sera was separated from the red cells by centrifugation at 5,000 rpm for 5 min. Sera samples were stored at −80°C in 1.5 ml microcentrifuge tubes at the Arbovirology Laboratory of the Dr. Hideyo Noguchi-Regional Research Center of the Autonomous University of Yucatan.

### Entomological survey

Entomological surveillance was carried out in 20 households in each locality to assess the presence of mosquitoes. Surveillance of the immature stages was performed once a month by reviewing natural and artificial breeding sites located inside the households and in peridomicile areas. Plastic waste that could contain water (bottle caps, bottles, plastic bags, etc.) and plastic containers used for water storage were considered as artificial breeding sites. Larvae and pupae were completely removed from the breeding sites with 7 ml disposable Pasteur pipettes and placed in plastic bottles labeled with the collection data. Adult mosquitoes were collected between 08:00 and 13:00 using backpack aspirators (Prokopack Aspirator^®^, models 1419, John W. Hock company). Each house was sampled on for three consecutive days per month, following the methodology described previously (26). The aspiration was done with particular attention to dark and humid places where the mosquitoes rest inside houses and their surroundings. Captured mosquitoes were kept in a cold chain in containers labeled with the date, house number and location, and were transported to the Arbovirology Laboratory of the Dr. Hideyo Noguchi-Regional Research Center of the Autonomous University of Yucatan. The morphological identification of the mosquitoes was carried out with a stereoscope (Carl Zeiss Microlmaging^®^, Germany) and dichotomous morphological keys (27). Since *Ae. aegypti* and *Cx. quinquefasciatus* are major flavivirus vectors in the area only these species were included for Zika detection in this study. Minimum infection rates (MIR: number of positive pools/total number of mosquitoes tested × 1,000) were estimated for these mosquito species for each community.

### RNA extraction

For RNA extraction, vertebrate samples were arranged into pools by season, species, and household. RNA was extracted with the Quick-RNATM Viral Kit (Zymo Research) according to the manufacturer's instructions.

For mosquitoes, clusters of *Ae. aegypti* and *Cx. quinquefasciatus* were homogenized with 300 μl of L-15 cell culture medium (Leibovitz-15) in 1.5 ml microcentrifuge tubes and subsequently centrifuged at 5,000 rpm for 5 min. 50 μl of the supernatant was then used for RNA extraction following the specifications of the Quick-RNATM Viral Kit (Zymo Research).

### Molecular flavivirus detection

Prior to the molecular detection of ZIKV, a preliminary screening was carried out to determine the presence of flavivirus in the collected samples. Reverse transcription (RT) was performed with 9.5 μl of RNA incubated with 0.5 μl of random primers (20 μg) (Promega) at 70°C for 5 min and subsequently at 4°C for 5 min. After this, 10 μl of the reaction mix [4 μl 5X Green buffer, 2 μl MgCl2 (25 mM), 1 μl dNTPs (10 μM), 1 μl GoScript™ Reverse Transcriptase (160 u) (Promega), and 2 μl nuclease-free water] were added and incubated for 5 min at 25°C, 1 h at 42°C and 5 min at 70°C. A Flavivirus heminested PCR was then performed according to previously reported protocols and primers that target a 251 bp region of the NS5 (28).

### Molecular Zika virus detection

Serum samples, which tested positive for flavivirus, were then assayed for ZIKV RNA by quantitative reverse transcriptase-polymerase chain reaction (qRT-PCR) with a total reaction volume of 10 μl with SoAdvanced Universal SYBR Green Supermix (Bio-Rad Laboratories, Hercules, CA) and Zika virus-specific primers previously reported (ZIKV-F: 5'-AGGATCATAGGTGATGAAGAAAAGT-3' and ZIKV –R:5'-CCTGACAACACTAAGATTGGTGC-3') (25). The primers target a 116 bp conserved region located between the NS5 and 3'UTR genes of ZIKV and do not amplify other related flaviviruses. The amplification conditions for the qRT-PCR were as follows: 42°C for 5 min, 95°C for 10 s and 40 cycles of 95°C for 5 s, 60°C for 34 s. RNA from other flavivirus (Dengue virus, West Nile virus and yellow fever virus) were also tested to evaluate the assay specificity. The limit of detection is 1 PFU/mL in ZIKV RNA extracts (CT value ≤ 34) as determined by previous reports (29).

### Sequencing

Positive PCR products were purified with DNA Clean and Concentrator^TM^ (Zymo research). PCR products with a concentration ≥5 ng/μl were sequenced in both directions by the Sanger method at the Biotechnology Institute of the National Autonomous University of Mexico to confirm positivity and rule out contamination. Obtained sequences were analyzed with the Sequencher version 4.1.4 software and subsequently compared with the National Center for Biotechnology Information (NCBI) database using the Basic Local Alignment Search Tool (BLAST) to determine their identity.

### Data analysis

Responses from the vector and disease knowledge surveys were organized into contingency tables and analyzed using the chi-square statistical test. If more than 25% of the cells had values lower than the expected value, Fisher's exact test was used to analyze the frequencies. The negative binomial regression model was used to estimate the risk of potential vectors based on the occurrence of *Ae. aegypti*. This model is used to make predictions when the data are overdispersed (α > 0) and the variance is higher than the mean (30). The number of *Ae. aegypti* females was used as the dependent variable because it represents an indicator of the potential epidemiological risk for arbovirus infection and transmission.

We also used the binary logistic regression model with the data on the presence and absence of the immature stages of *Ae. aegypti*. In both models, the predictor variables were the responses from the surveys on social marginalization, disease knowledge, vector, and presence of backyard animals.

The number of females of *Ae. aegypti* per house and between communities was analyzed with the U-Mann-Whitney non-parametric test for independent groups because they did not present normal distribution and homogeneity of variance. Statistical analyzes were performed using IBM SPSS version 22 statistical package for Windows (IBM Corporation, Armonk, NY). Data was considered statistically significant when bilateral *P* ≤ 0.05.

To determine the variables that influence the presence of ZIKV in humans, we tested seven predictor variables with a binary logistic regression model. The variables included in the model were: the inhabitants' age and gender; the house's construction (roof and wall material); kitchen location (inside or outside the house); bathroom location (inside or outside); and seasonality (dry or rainy). If there was an infection, the response variable was coded as 1, and if there was no infection, it was coded as 0. We split the dataset into training and test sets. We used 70% of the data for model fitting (training set) and the remaining data for validation (test set). The analysis of the logistic regression model was performed using the R statistical programming language version 4.0.2 and the caret package for the data splitting and the training set. The generalized linear model (GLM) function, with the family = “binomial” option, was used to estimate regression parameters and perform the data analysis. The likelihood ratio test was used to assess the significance of the overall model with k predictors. Z statistics and p-values were given in the R regression output. Small p-values (≤0.05) indicated the corresponding predictors were significant. The fitted models were evaluated by the receiver operating characteristic (ROC) curve. The ROC curve is a line plot that is drawn between the sensitivity and (1 – specificity). The graph is then used to generate the AUC value. An AUC value of >0.70 indicates a good model.

## Results

### Social marginalization

A questionnaire was applied to identify social marginalization in two Mayan communities. From the data, we determined that 87.80% of our participants were female (36/41). The average age of household heads was 42-year-old. More women than men participated in our study because females were at home during the time of the interview.

**Figure 2:**
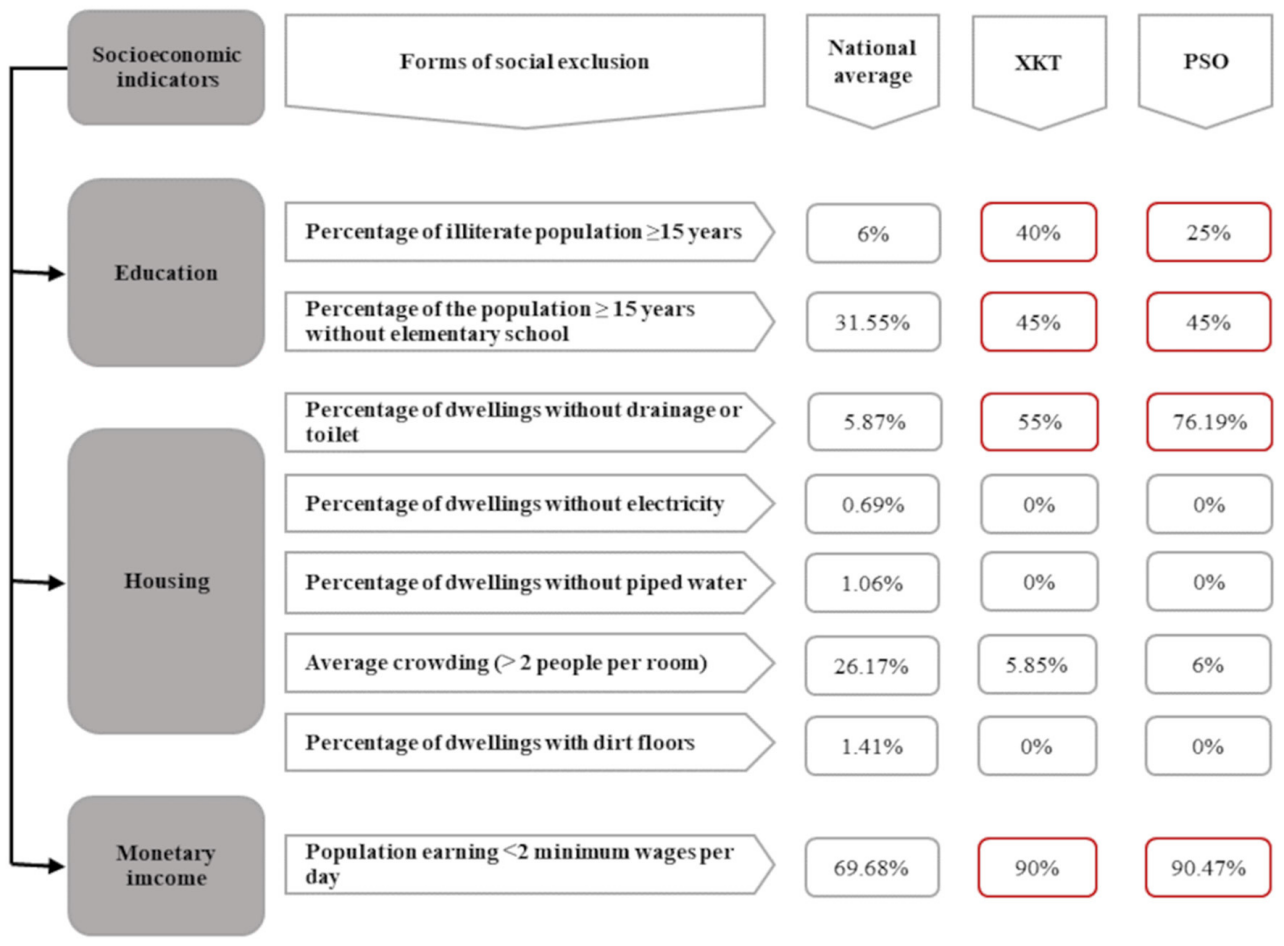
Conceptual scheme of social marginalization of two Mayan communities of Yucatan, México. Here we compared the national average of each of the exclusion forms, design for the National Population Council of Mexico to measure social marginalization (20), with the two Mayan communities studied. We can observe in red the collected data that exceeds the national average. XKT, Xkalakdzonot; PSO, Paraiso.

The results confirmed social vulnerability of the communities, as four out of the eight forms of social exclusion were observed to exceed the national average (Figure 2). The percentage of illiterate people varied according to the community. In XKT, 40% (8/20) of the participants were illiterate, while in PSO the percentage was 25% (5/20). The percentage of participants with only elementary school education was 15% (3/20) for XKT and 30% (6/20) for PSO. Only 10% of the interviewed participants in both communities (2/40) finished high school.

Overcrowding was observed in both communities with an estimated average of 5.85 people per room for XKT and 6 for PSO. In terms of monetary income, 90% of families live with less than two minimum wages in both communities (Figure 2) (In Mexico, the minimum wage for 2016 was $73.04 Mexican pesos per day, equivalent to $3.94 US dollars per day).

A hundred per cent of the surveyed houses have electricity service, piped water, and concrete floors in at least one room. Despite having piped water there is a lack of proper infrastructure, W.C facilities are not adequate and severe water overflows from the sewage system is common (100%). The lack of proper sewage disposal in houses of both communities was high; 55% (11/20) of the dwellings in the community of XKT and 76.19% (16/21) in PSO didn't have septic tanks. In addition, 95% of families enrolled in the study commonly stored water in barrel containers despite having water pipelines. It was noted that water supply is not constant, limiting the availability of the resource in the communities. Regarding waste management, neither of the communities has proper waste management systems. Accumulation of non-organic waste, mainly plastic, such as bags, bottles, and old electronic appliances carcasses in the yards was observed. There was no domestic telephone line or internet in any of the houses included in this study. All the enrolled families declared that they do not have access to piped gas and 100% of them use firewood for cooking.

### Entomological survey

In XKT, 1,508 possible artificial breeding sites were identified, of which 755 contained water. In PSO, 736 of the 1,410 possible artificial breeding sites observed had water. The presence of larvae and pupae was commonly observed in disposable plastic containers like buckets, metal barrels and small natural puddles.

In both communities, the cumulative number of mosquito species was 15. Eleven were in the immature stage and 12 in the adult stage (Supplementary Table 1). Greater abundance of female *Culex* spp. was observed. The most abundant species where *Culex quinquefasciatus* (*n* = 5,880), followed by *Aedes aegypti* (*n* = 765), *Culex nigripalpus* (*n* = 416) and *Aedes taeniorhynchus* (*n* = 196). The less abundant species were *Aedes cozumelensis* (*n* = 22)*, Toxorhynchites theobaldi* (*n* = 8), *Aedes trivitattus* (*n* = 7), *Anopheles albimanus* (*n* = 5), *Culex declarator* (*n* = 5), *Culex coronator* (*n* = 2), *Psorophora ferox* (*n* = 1) and *Limatus durhamii* (*n* = 1). The abundance of *Ae. aegypti* and *Cx. quinquefasciatus* in both communities is shown in Table 1.

**Table 1:** Abundance of mosquitoes of the species *Ae. aegypti* and *Cx. quinquefasciatus* captured in two Mayan communities of Yucatan, Mexico.

	**Xkalakdzonot (XKT)**			**Paraíso (PSO)**		
	**Dry season**	**Rainy season**	**Total**	**Dry season**	**Rainy season**	**Total**
*Ae. aegypti*	30	152	182	45	538	583
*Cx. quinquefasciatus*	1,255	2,435	3,690	412	1,778	2,190

The average number of captured females of *Ae. aegypti* was 29.2 per house in PSO, while in XKT it was 9.1 per house, resulting in a significant difference (Z = −2.80, *P* = 0.005). Regarding *Cx. quinquefasciatus*, the average number of captured females per house was 109.5 and 184.5 in PSO and XKT, respectively. The negative binomial regression model was significant (X^2^ = 12.698, g.l. = 5, *P* = 0.026). The presence of chickens in the backyard (incidence rate = 1.06) and the overcrowding of humans in dwellings (incidence rate = 1.13) were the most important factors for the presence of *Ae. aegypti*. The logistic regression model estimated that the presence of hens (odds ratio = 27.41) and septic tanks (odds ratio = 6.64) are risk factors for the presence of larvae and pupae of *Ae. aegypti* in houses of both communities.

### Backyard animals

Throughout the study, a total of 1,055 backyard animals were sampled in both communities. Of these, 791 (74.97%) were chickens, 141 (13.36%) pigs, and 123 (11.65%) turkeys. During the dry season, chickens and turkeys were more abundant in both XKT and PSO (Table 2). In XKT, pig rearing was observed throughout the year, with a higher number of pigs during the rainy season (Table 2). At the time of the study there was no pig rearing in PSO, since this activity occurs intermittently in the community.

**Table 2:** Seasonal inventory of backyard animals, percentage of households that breeds them and average number of animals per household of two Mayan communities of Yucatan, Mexico.

	**Xkalakdzonot (XKT)**						**Paraiso (PSO)**					
	**Dry season**			**Rainy season**			**Dry season**			**Rainy season**		
	**n/N**	**%**	$\bar{x}$	**n/N**	**%**	$\bar{x}$	**n/N**	**%**	$\bar{x}$	**n/N**	**%**	$\bar{x}$
		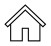	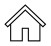		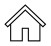	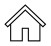		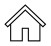	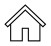		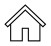	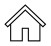
Chickens (*Gallus gallus*)	348/555	90%	17.4	207/555	65%	10.3	135/236	85%	6.8	101/236	70%	5
Turkeys (*Meleagris gallopavo*)	34/59	30%	1.7	25/59	35%	1.3	60/64	55%	3	4/64	5%	0.2
Pigs (*Sus scrofa domestica*)	59/141	85%	2.9	82/141	70%	4.1	-	-	-	-	-	-

n, number of animals sampled per season; N, total number of animals sampled; % 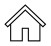, percentage of household that breeds chicken, turkeys and/or pigs; x 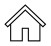, average number of chickens, turkeys and/or pigs per household.

### Vertebrates Zika virus detection

#### Pigs

**Figure 3:**
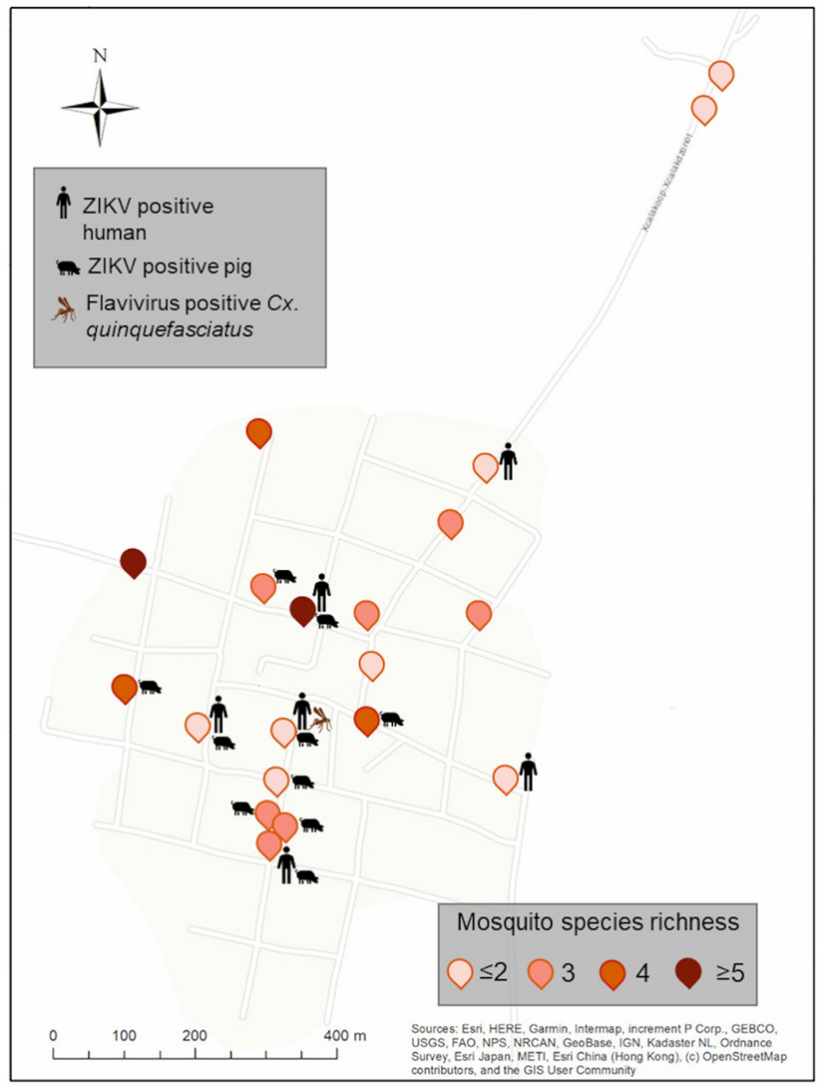
Spatial distribution of ZIKV-positive pools of vertebrates and flavivirus positive mosquitoes from Xkalakdzonot (XKT), Yucatan, Mexico. Positive households include the icons of vertebrates or mosquito pools positive in each dwelling. Mosquito species identity for each household is shown in Supplementary Table 2. Map coordinates were removed to protect the privacy of the patients involved.

Pig farming was observed exclusively at XKT. In this community a total of 141 individuals were sampled, 59 during the dry season and 82 during the rainy season (Table 2). Ten out of the 32 (31.2%) pools analyzed, were positive for ZIKV, all belonging to samples collected in the dry season (Table 3). The ages of the pigs included in the positive pools ranged from 1 month up to 3 years, being 5 of the pools ≤ 8 months and the rest ≥ 1.5–3 years. The distribution within the community (XKT) of the positive pools is shown in Figure 3. No positive animals were recorded during the rainy season.

**Table 3:** Positive vertebrates and prevalence of infection in two Mayan communities of Yucatan, Mexico.

		**Sampled**	**Pools tested**	+ individuals^*^	**Prevalence**
Xkalakdzonot (XKT)	Pigs (*Sus scrofa domestica*)	141	32	10 (ZIKV)	7.09 (ZIKV)
	Humans (*Homo sapiens*)	184	-	13 (ZIKV)	7.07 (ZIKV)
Paraíso (PSO)	Humans (*Homo sapiens*)	219	-	5 (ZIKV)	2.28 (ZIKV)

* In the pools of pigs that tested positive for ZIKV, at least one individual was considered positive.

#### Poultry

Chickens and turkeys were collected in both communities. The chicken population was higher in XKT than in PSO, while the turkey population was higher in PSO than in XKT (Table 2). In XKT, 614 (555 chickens and 59 turkeys) poultry were sampled, while in PSO, 300 (236 chickens and 64 turkeys) poultry were sampled. All animals were adults. A total of 116 poultry pools were tested (73 XKT and 43 PSO), none of which tested positive for ZIKV.

#### Humans

Twenty houses per community were sampled, obtaining a total of 416 human blood samples (197 from XKT and 219 from PSO). Of the samples analyzed, 18 (4.32%) were positive for ZIKV, 9 corresponded to men and 9 to women; all samples were collected during the rainy season. XKT presented a higher frequency of positive cases (13/197) in contrast to PSO (5/219). The age of positive individuals ranged from 1 to 80 years. In XKT, the frequency of positive cases was higher among the young (1–18 years old), representing 61.5% (8/13) of the cases. The rest were distributed between 19 and 40 years (15.3%, 2/13) and older than 50 years (23%, 3/13) (Table 4). In PSO 40% (2/5) of the positive cases were under 18 years old, 40% (2/5) between 20 and 40 years old, and 10% above 40 years old (1/5) (Table 4). Of the positive individuals, one was febrile, while the rest had no symptoms.

**Table 4:** Zika virus-positive humans in two Mayan communities in Yucatan, Mexico.

	**+ZIKV Household**	**+ ZIKV/sampled**	**Age**	**Sex**
Xcalakdzonot (XKT)	C3	3/11 (27.2%)	21	F
			1	M
			1	M
	C7	3/9 (33.3%)	4	M
			6	M
			77	F
	C9	2/4 (50%)	51	F
			3	M
	C18	1/3 (33.3%)	18	F
	C19	1/5 (20%)	25	F
	C20	3/9 (33.3%)	12	M
			3	M
			67	F
Paraíso (PSO)	C9	1/4 (25%)	43	M
	C16	2/10 (20%)	34	F
			20	F
	C19	1/1 (100%)	9	F
	C20	1/4 (25%)	9	M

+ ZIKV/sampled, ZIKV positive individuals / Individuals sampled per household; F, Female; M, Male.

**Figure 4:**
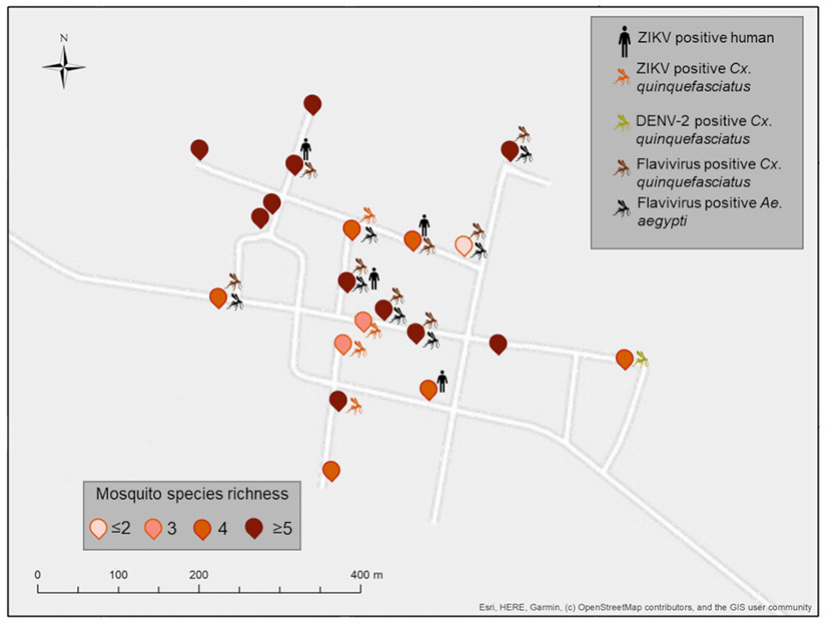
Spatial distribution of ZIKV-positive pools of vertebrates and mosquitoes from Paraiso (PSO), Yucatan, Mexico. Positive households include the icons of vertebrates or mosquito pools positive in each dwelling. Mosquito species identity for each household is shown in Supplementary Table 2. Map coordinates were removed to protect the privacy of the patients involved.

Ten of the households studied, six in XKT and four in PSO, had at least one positive individual for ZIKV (Figures 3, 4). The bathroom location was the only significant predictor that affected the likelihood of having a positive Zika virus case in humans as correlates with high number of mosquitoes (*p* ≤ 0.05). In Table 5, regression coefficients and p-values are shown. The regression coefficient *B*1 is 2.4849. The probability of finding a positive sample for Zika increases by 11.99% in houses with an outdoor bathroom compared to those with an indoor bathroom. The probability was obtained by exp (2.4849) = 11.99%. In the training dataset, the logistics model correctly classified 97.60% of all observations. The area under the curve (AUC) for the training datasets was 0.95.

**Table 5:** Parameters of the logistic regression model.

	**Estimate**	**Std. Error**	**Z- value**	***P*** **-value**
(Intercept)	−25.81	18,725.03	−0.001	0.99
Bathroom location Outside	2.48	0.84	2.95	0.003^*^

* P < 0.05.

#### Mosquitoes

Molecular analysis for the detection of Flavivirus was only performed on *Cx. quinquefasciatus* and *Ae. aegypti*, since they were the most abundant in both communities and are known vectors of arboviruses. 6,645 mosquitoes were collected, of which 765 were *Ae. aegypti* and 5,880 *Cx. quinquefasciatus* (Table 1). The average of *Cx. quinquefasciatus* and *Ae. aegypti* captured per house was 184.5 and 9.1, respectively for XKT, while in PSO the average per house was 109.5 for *Cx. quinquefasciatus* and 29.2 for *Ae. aegypti*.

Mosquitoes were clustered into pools according to species, season, and backyard, so the number of mosquitoes in each group varies. Based on this, 381 pools were obtained for both communities, 270 from *Cx. quinquefasciatus* (141 from XKT and 129 from PSO) and 111 from *Ae. aegypti* (37 from XKT and 74 from PSO). Flavivirus RNA was detected in 43/381 pools analyzed (12/111 *Ae. aegypti* and 31/270 *Cx. quinquefasciatus* pools). For XKT 2/141 pools of *Cx. quinquefasciatus* were positive (MIR = 0.54); while in PSO 12/74 of *Ae. aegypti* (MIR = 20.58) and 29/129 of *Cx. quinquefasciatus* were positive (MIR = 13.24) (Table 6).

**Table 6:** Pools and MIR of *Ae. aegypti* and *Cx. quinquefasciatus* positive for Flavivirus in two Mayan communities of Yucatan, Mexico.

	**Xkalakdzonot (XKT)**			**Paraíso (PSO)**
	***Ae. aegypti***	***Cx. quinquefasciatus***	***Ae. aegypti***	***Cx. quinquefasciatus***
Positive pools	0/37	2/141	12/74	29/129
Minimum infection rate	–	0.54	20.58	13.24

Ten PCR products from mosquitoes were sequenced, four from *Ae. aegypti* and six from *Cx. quinquefasciatus*. Three of the six sequences from *Cx. quinquefasciatus* were confirmed as ZIKV, with coverage and identity percentages >95% (GenBank accession numbers: OP431392, OP431393 and OP431394). One of the six sequences also showed high percentages of coverage and identity (>95%) with DENV-2, however the size of the recovered sequence was <200 bp. The sequences obtained from *Ae. aegypti* were of low quality, so their identity was not determined.

## Discussion

In this study, we demonstrated the simultaneous circulation of Zika virus at the animal-human interface in areas where traditional farming is practiced, proposing the maintenance of enzootic and epidemic transmission cycles. This phenomenon is exacerbated by poor socioeconomic development and marginalization of communities where traditional farming is practiced (31), suggesting a high-risk transmission of zoonotic diseases to vulnerable populations (32). In addition to the health impact, the social and economic burden of Zika-associated disease are considerable on vulnerable population. ZIKV caused the average yearly loss of over 44,000 DALYs (disability-adjusted life year) globally between 2010 and 2019 (20), due to out-of-pocket medical care, especially to cover those expenses associated to the impact of congenital Zika syndrome cases.

A study conducted in Vietnam found a significant association between garbage accumulation, backyard animal husbandry, latrine use and, increased prevalence of DENV antibodies in susceptible individuals (33). Similar to what we observed in the Yucatan communities, poor infrastructure, water accumulation, and lack of waste disposal, allow mosquitos' development and proliferation, thus increasing arboviruses incidence. Also, the lack of sanitation and infrastructure for wastewater and solid waste disposal, irregular access to piped water and, overcrowding, are important factors favoring vector-borne diseases transmission (34).

It has been demonstrated that heterogeneity of mosquito populations favors viral genetic modifications (35). In addition, depending on feeding preferences, mosquitoes serve either as vectors among humans, non-human vertebrates, or as bridging vectors between animals and humans. In this regard, *Cx. quinquefasciatus* was the most abundant mosquito in our study areas, being an opportunistic species that feeds on birds and mammals, including humans (36, 37). The second most abundant species, *Ae. aegypti*, is partially opportunistic with a greater preference for human hosts, making them both, the most important arbovirus vectors worldwide (37). In the studied communities, both species were positive for flaviviruses; however, only ZIKV and DENV-2 were detected in pools of *Cx. quinquefasciatus*.

Despite *Cx. quinquefasciatus* is a recognized vector of flaviviruses such as West Nile virus (WNV), Japanese encephalitis virus (JEV), and Saint Louis encephalitis virus (SLEV) (36), its role in ZIKV transmission is still uncertain. The rapid spread and severity of the ZIKV epidemic that occurred in the Americas in 2015–2016 has raised suspicions that *Cx. quinquefasciatus* is an additional vector of this virus (38, 39). Some studies suggest that *Cx. quinquefasciatus* is refractory to ZIKV infection and disseminates poorly outside the midgut barrier (39–41). On the other hand, experimental infections have demonstrated effective ZIKV dissemination in the midgut, salivary glands, and saliva (42) and have even described vertical transmission (43) in this mosquito species. There is also evidence of ZIKV in mosquitoes caught in a region of high Zika incidence in Brazil, from which the virus could be isolated in Vero cells (42). Similarly, in Jalisco, Mexico, the presence of ZIKV has been reported in the midgut, salivary glands, and entire body *of Cx. quinquefasciatus* and other mosquito species of the same genus collected in the field (44). Despite the vector competence and field infection rate, current data is not completely conclusive about its relative role, we can hypothesize its potential enzootic or bridge vector capacity for Zika virus due to its widespread distribution, high local abundance, and opportunistic feeding behavior.

*Ae. aegypti* is the major vector of flaviviruses transmitted by mosquitoes, especially DENV and ZIKV (45). Unfortunately, it was not possible to determine the presence of flaviviruses in this species. However, recent studies in Yucatan, Mexico, have shown that *Ae. aegypti* is infected with DENV (serotypes 1 and 4) and ZIKV (46–48). Although *Ae. aegypti* is a far more competent vector for ZIKV transmission, local studies have also shown low ZIKV transmission by urban *Ae. aegypti* from Yucatán (49). This suggests the involvement of alternative or lesser-known vectors that may be involved in ZIKV transmission between animals and humans. A weakness of our study was the limits on the molecular analysis of flaviviruses only on *Cx. quinquefasciatus* and *Ae. aegypti*, because a more comprehensive look at the other mosquito species could have given us a hint into the dynamics that occur in these backyard environments. In addition, vector competence studies need to be conducted to identify the mosquito species that can more efficiently transmit viruses to humans and domestic animals in these particular populations.

Zika prevalence was higher in XKT than in PSO. Most positive cases in XKT were in an age group of 1–18 years. Similar results were described in a cohort study in different cities of Yucatan, Mexico, in 2015–2016, where >70% of the studied individuals had seroprevalence for arboviruses such as DENV, ZIKV, and chikungunya (50). The higher prevalence of Zika virus in humans (7.07 vs. 2.28) was also reflected in non-human vertebrates in XKT compared with PSO (Table 3). Although both communities have year-round poultry rearing, one major difference is associated with the presence of pigs. Monogastric species are common in backyard rearing because they grow rapidly and have a high reproductive rate (51), resulting in continuous reintroduction of naive individuals into the population, thus maintaining viral transmission cycles.

A high prevalence of ZIKV was found in the pig population of XKT. Experimental studies have shown that ZIKV can infect pigs, depending on the method of inoculation (52, 53). Natural ZIKV infection in pigs has been previously reported in Yucatan (54). Although no clinical signs were observed in these animals, serologic data showed that pigs temporally and spatially associated with humans had monotypic neutralizing antibodies to Zika virus. Although some experimental studies showed no pathologic signs or antibody responses, naturally exposed pigs have the potential to establish enzootic cycles, potentially transmitting these viruses to other domestic or wild animals (55) or susceptible humans.

No ZIKV-positive poultry were detected in any of the communities. There is limited information on natural mosquito-borne flavivirus infections in poultry. Most studies have focused on viruses belonging to the Japanese encephalitis serogroup, mainly WNV, JEV, SLEV, and Usutu (56–60). In the Americas, chickens in Puerto Rico have been used as sentinels for WNV (61); neutralizing antibodies to WNV and SLEV have been detected in poultry from Chiapas, Mexico (62); and seroconversion and low viremia have been observed in hens experimentally inoculated with WNV (63). As for ZIKV, experimental studies have shown that it is permissive in embryonic chicken cells and chicken embryos, in which high mortality and central nervous system abnormalities have been observed (64–66). In contrast, adult chickens with intact immune systems showed no pathological signs, viremia, or neutralizing antibodies after inoculation with ZIKV (64). Despite the experimental data, a study conducted during active ZIKV transmission in Brazil at an urban/forest interface showed monotypic responses to ZIKV in chickens (22). This is interesting because many of the chickens studied coexisted with ZIKV-positive pigs and humans, which may indicate that active infection had occurred in these vertebrates before the sampling period, but further serologic analysis and continuous molecular testing would be needed to clarify this field observation.

A weakness of this work is the lack of virus isolation and proper characterization of the viruses found in the animal and human populations. This potentially may have given us insight into arboviral diversity at the study sites to understand possible spillover effects and adaptation of viruses to different vertebrate populations (67).

It is important to emphasize that the previously mentioned experimental studies were conducted under different conditions that may affect or bias the result, such as the origin of the mosquito colonies or animals used for the study, the virus strain and concentration to which they were exposed, and the mode of inoculation (39, 68, 69). This last aspect is important when it comes to mosquito-borne viruses because it has been observed that vertebrates experimentally exposed *via* mosquitoes or in combination with mosquito saliva have a higher infection rate compared with needle inoculations (70, 71). All these aspects may reflect different scenarios between what is observed in laboratory situations and what occurs in naturally exposed populations.

## Data availability statement

The datasets presented in this study can be found in online repositories. The names of the repository/repositories and accession number(s) can be found in the article.

## Ethics statement

The studies involving human participants were reviewed and approved by Research Ethics Commission under the supervision of Hospital General de Mexico Dr. Eduardo Liceaga (Registry key: DI/14/ 404D/05/050). Written informed consent to participate in this study was provided by the participants' legal guardian/next of kin. The animal study was reviewed and approved by University Autonomous of Yucatan (CB-CCBA-I-2017-006). Written informed consent was obtained from the owners for the participation of their animals in this study.

## Author contributions

LL-A performed field work, sample processing, and wrote the original draft. HZ analyzed data and contributed to the mapping of the communities. EZ-M performed field work, sample processing, and bioinformatic tools. CB-B performed the mosquito identification and data analysis. GR-S design and conducted socioeconomic surveys and social work in the communities. DN-A, T-CO, IP-B, DR, and GS critically reviewed the manuscript and provided enriching ideas to the final version of the manuscript. CM-W contributed to the conception and design of the study and secure funding for the research. All authors read and approved the final manuscript.

## Funding

This study was supported in part by Consejo Nacional de Ciencia y Tecnología de México (CONACyT)–Problemas nacionales (Grant No. PDCPN 2014-247005) and in part by CONACyT- Paradigmas y controversias de la ciencia (Grant No. 320559).

## Conflict of interest

The authors declare that the research was conducted in the absence of any commercial or financial relationships that could be construed as a potential conflict of interest.

## Publisher's note

All claims expressed in this article are solely those of the authors and do not necessarily represent those of their affiliated organizations, or those of the publisher, the editors and the reviewers. Any product that may be evaluated in this article, or claim that may be made by its manufacturer, is not guaranteed or endorsed by the publisher.
