# Supplementary material for: Molecular survey of Zika virus in the animal-human interface in traditional farming
Source: Front Vet Sci. 2022 Nov 25;9:1057686. doi: 10.3389/fvets.2022.1057686 (PMC9732010; doi:10.3389/fvets.2022.1057686)
Supplement: Supplementary file 1 [file Data_Sheet_1.docx]

Supplementary Material

# Supplementary Tables

| **S1 Table.** Abundance and presence of immature stages of mosquitoes in two Mayan communities of Yucatan, Mexico. | | | | |
| --- | --- | --- | --- | --- |
|  | Xkalakdzonot (XKT) | | Paraíso (PSO) | |
| Mosquito species | Mosquito abundance | Presence of immature stages | Mosquito abundance | Presence of immature stages |
| *Aedes* (*Stegomyia*) *aegypti* (Linnaeus) | 182 | √ | 583 | √ |
| *Aedes* (*Howardina*) *cozumelensis* Díaz Nájera | 13 | √ | 9 |  |
| *Aedes* (*Ochlerotatus*) *taeniorhynchus* (Wiedemann) | 7 |  | 189 |  |
| *Aedes* (*Ochlerotatus*) *trivittatus* (Coquillett) | 2 | √ | 5 |  |
| *Aedes* (*Protomacleaya*) *podographicus* Dyar and Knab | 0 | √ | 0 |  |
| *Anopheles* (*Nyssorhynchus*) *albimanus* Wiedemann | 0 |  | 5 |  |
| *Culex* (*Culex*) *coronator* **s.l.** Dyar and Knab | 1 | √ | 1 | √ |
| *Culex* (*Culex*) *declarator* Dyar and Knab | 0 |  | 5 |  |
| *Culex* (*Culex*) *nigripalpus* Theobald | 6 |  | 410 | √ |
| *Culex* (*Culex*) *quinquefasciatus* Say | 3690 | √ | 2190 | √ |
| *Culex* (*Culex*) *stigmatosoma* Dyar | 0 |  | 0 | √ |
| *Culex (Phenacomyia) corniger* Theobald | 0 | √ | 0 |  |
| *Limatus durhamii* Theobald | 1 | √ | 0 |  |
| *Psorophora* (*Janthinosoma*) *ferox* (von Humboldt) | 0 |  | 1 |  |
| *Toxorhynchites* (*Lynchiella*) *theobaldi* (Dyar and Knab) | 0 | √ | 8 | √ |

| **S2 Table.** Distribution among households of adult mosquito species in two Mayan communities of Yucatán, Mexico. | | | | | | | | | | | | | | | | | | | | |
| --- | --- | --- | --- | --- | --- | --- | --- | --- | --- | --- | --- | --- | --- | --- | --- | --- | --- | --- | --- | --- |
| Mosquito species | Households | | | | | | | | | | | | | | | | | | | |
|  | 1 | 2 | 3 | 4 | 5 | 6 | 7 | 8 | 9 | 10 | 11 | 12 | 13 | 14 | 15 | 16 | 17 | 18 | 19 | 20 |
| *Aedes* (*Stegomyia*) *aegypti* (Linnaeus) | P | P | X P | X P | X P | X P | X P | X P | X P | X P | X P | X P | X P | P | X P | X P | X P | X P | P | X P |
| *Aedes* (*Howardina*) *cozumelensis* Díaz Nájera |  | P |  |  | P | X | P | X |  |  |  |  | X |  |  | X |  |  |  |  |
| *Aedes* (*Ochlerotatus*) *taeniorhynchus* (Wiedemann) | P | P |  | P | X P | P | X P | P | P | X P | P | X P | X P | X P | P | P | P | X | P | P |
| *Aedes* (*Ochlerotatus*) *trivittatus* (Coquillett) |  |  |  |  |  |  |  |  |  |  |  | X P |  |  |  |  | P |  |  | P |
| *Anopheles* (*Nyssorhynchus*) *albimanus* Wiedemann |  |  | P |  |  | P |  |  |  |  |  |  | P | P |  |  |  |  |  | P |
| *Culex* (*Culex*) *coronator* **s.l.** Dyar and Knab |  |  |  |  |  |  |  |  |  |  |  |  | P |  |  |  | X |  |  |  |
| *Culex* (*Culex*) *declarator* Dyar and Knab |  |  |  |  |  |  | P |  |  |  |  | P |  |  |  |  | P |  |  |  |
| *Culex* (*Culex*) *nigripalpus* Theobald | P | P |  | X | P | P | X P | P | P | P | P | P | X P | P | P | P | P |  | P | P |
| *Culex* (*Culex*) *quinquefasciatus* Say | X P | X P | X P | X P | X P | X P | X P | X P | X P | X P | X P | X P | X P | X P | X P | X P | X P | X P | X P | X P |
| *Culex* (*Culex*) *tarsalis* Coquillett |  |  |  |  |  |  | X |  |  | X |  |  |  | X |  |  |  |  |  |  |
| *Limatus durhamii* Theobald |  |  |  |  |  |  | X |  |  |  |  |  |  |  |  |  |  |  |  |  |
| *Psorophora* (*Janthinosoma*) *ferox* (von Humboldt) |  |  |  |  |  |  |  |  |  |  |  |  |  |  |  | P |  |  |  |  |
| *Toxorhynchites* (*Lynchiella*) *theobaldi* (Dyar and Knab) |  | P |  |  | P |  | P |  |  |  | P | P |  |  |  |  |  |  |  |  |
| X: Xkalakdzonot (XKT); P: Paraiso (PSO) | | | | | | | | | | | | | | | | | | | | |

**S3 Table.** Household distribution of humans, mosquitoes, and animals positive for Zika Virus

|  | Household | ZIKV-positive | | |
| --- | --- | --- | --- | --- |
|  |  | Humans  (*Homo sapiens*) | Mosquitoes | Pigs  (*Sus scrofa domestica*) |
| Xkalakdzonot (XKT) | C3 | √^R^ |  |  |
|  | C4 |  |  |  |
|  | C7 | √^R^ |  | √^D^ |
|  | C8 |  |  | √^D^ |
|  | C9 | √^R^ |  |  |
|  | C10 |  |  | √^D^ |
|  | C11 |  |  |  |
|  | C13 |  |  |  |
|  | C14 |  |  | √^D^ |
|  | C15 |  |  | √^D^ |
|  | C16 |  |  | √^D^ |
|  | C17 |  |  | √^D^ |
|  | C18 | √^R^ |  | √^D^ |
|  | C19 | √^R^ |  | √^D^ |
|  | C20 | √^R^ | √^Rc^ | √^D^ |
| Paraiso (PSO) | C2 |  | √^Rc^ |  |
|  | C3 |  | √^RDc^ |  |
|  | C4 |  | √^Rc^ |  |
|  | C5 |  | √^DaRac^ |  |
|  | C6 |  | √^Rac^ |  |
|  | C8 |  | √^DcRc^ |  |
|  | C9 | √^R^ |  |  |
|  | C10 |  | √^DaRa^ |  |
|  | C15 |  | √^DaRc^ |  |
|  | C16 | √^R^ | √^DaRac^ |  |
|  | C17 |  | √^Rac^ |  |
|  | C18 |  | √^DcRa^ |  |
|  | C19 | √^R^ | √^Rc^ |  |
|  | C20 | √^R^ | √^Rc^ |  |

R = rainy season; D = dry season; a = *Ae. aegypti*; c = *Cx. quinquefasciatus*
